# Miniaturization and Automation of a Human In Vitro Blood–Brain Barrier Model for the High-Throughput Screening of Compounds in the Early Stage of Drug Discovery

**DOI:** 10.3390/pharmaceutics13060892

**Published:** 2021-06-16

**Authors:** Elisa L. J. Moya, Elodie Vandenhaute, Eleonora Rizzi, Marie-Christine Boucau, Johan Hachani, Nathalie Maubon, Fabien Gosselet, Marie-Pierre Dehouck

**Affiliations:** 1 Laboratoire de la Barrière Hémato-Encéphalique (LBHE), University Artois, UR 2465, F-62300 Lens, France; elisajimenezmoya@gmail.com (E.L.J.M.); eleonora.rizzi@univ-artois.fr (E.R.); mchristine.boucau@univ-artois.fr (M.-C.B.); johan.hachani@univ-artois.fr (J.H.); fabien.gosselet@univ-artois.fr (F.G.); 2 HCS Pharma, F-59120 Loos, France; elodie.vandenhaute@hcs-pharma.com (E.V.); nathalie.maubon@hcs-pharma.com (N.M.)

**Keywords:** blood–brain barrier (BBB), in vitro models, BBB permeability, screening assays, automated system, CNS drug screening, brain delivery

## Abstract

Central nervous system (CNS) diseases are one of the top causes of death worldwide. As there is a difficulty of drug penetration into the brain due to the blood–brain barrier (BBB), many CNS drugs treatments fail in clinical trials. Hence, there is a need to develop effective CNS drugs following strategies for delivery to the brain by better selecting them as early as possible during the drug discovery process. The use of in vitro BBB models has proved useful to evaluate the impact of drugs/compounds toxicity, BBB permeation rates and molecular transport mechanisms within the brain cells in academic research and early-stage drug discovery. However, these studies that require biological material (animal brain or human cells) are time-consuming and involve costly amounts of materials and plastic wastes due to the format of the models. Hence, to adapt to the high yields needed in early-stage drug discoveries for compound screenings, a patented well-established human in vitro BBB model was miniaturized and automated into a 96-well format. This replicate met all the BBB model reliability criteria to get predictive results, allowing a significant reduction in biological materials, waste and a higher screening capacity for being extensively used during early-stage drug discovery studies.

## 1. Introduction

Central nervous system (CNS) diseases are a relevant burden on healthcare systems, being one of the top causes of death worldwide, according to the World Health Organization (WHO) [1]. Brain disease incidences will continue growing due to the aging population increase [2]. Nevertheless, there are still a large number of unmet medical needs concerning the treatment of most CNS diseases [3]. Hence, within the global pharmaceutical market, the neuropharmaceuticals market is potentially one of the largest sectors aiming to increase and improve the average life expectancy [4]. However, the development of drugs for treating CNS disorders is a medically challenging and commercially risky field. Numerous medical research efforts are focused on finding drug candidates for CNS disease treatments, but as the brain is one of the more protected organs, few neurological disorders have fully benefited from pharmacotherapy. One of the main limitations for the treatment of neurological disorders is the difficulty to deliver drugs to the brain. This protection mainly comes from the blood–brain barrier (BBB), which is a selective barrier formed by the endothelial cells of cerebral microvessels. The BBB is essential to maintain brain homeostasis [5] but is also one of the major causes of failure for new CNS drug candidates to access the brain [4,6].

The probability of success in obtaining a marketing authorization is less than 7% [7]. Moreover, the time needed considering the clinical and regulatory phases is around 10.5 years, the longest compared to other therapeutic areas [8]. Despite these considerations, the designing of potential neuropharmacological drugs and innovative strategies for delivery to the brain [9,10,11,12] have significantly increased nowadays, from both the public and private sectors. For example, currently, many researches are focused on the development and utilization of nanoparticles (NP) as promise drug carriers, proposed as a solution for brain penetration [13]. Nevertheless, the hurdle of NP passage across the BBB, as well as the pharmacological and toxicological aspects of these nanomedicines [14], must be deeply studied in the early stages of drug discovery in order to reduce the risk of costly later failures in the clinical phases. Therefore, reliable methods for selecting the best candidates in the early preclinical phases are urgently needed [15]. However, the new drug and therapeutic strategy screenings are also very expensive and time-consuming, where in vivo screening remains applied at the preclinical testing stage. Much research effort is therefore directed toward the development of functional BBB in vitro models [16] that would make new faster available drugs for the following steps of validation.

There has been a great interest in the development of in vitro models to mimic the BBB permeability properties. Focusing on the concern of CNS drug research in the early stages discovery, these models can give good insight about the drug toxicity and the study of drug transport mechanisms through brain endothelial cells, giving an evaluation of the ability to access the brain. Moreover, cell culture systems decrease the use of animals in these preliminary phases of drug discovery, as well as for general academic purpose studies. Hence, the use of in vitro BBB models constitutes a relevant improvement in terms of the time, financial and ethical-saving aspects, where numbers of animals are greatly reduced for this very early stage of drug discovery.

Several static BBB in vitro models have been developed through the last decades, following different conformations like monocultures, cocultures and tricultures using different brain cell types and coming from different animal species donors either cultured from primary cells or immortalized cell lines, resulting in a large variation of experimental protocols, cell lines and model configurations [17]. More recently, microfluidics devices have also gained ground [18,19]. However, the BBB in vitro models field could greatly benefit from the development of high-throughput screening systems (HTS) to allow the study of a high numbers of drugs, compounds, nanoformulations or nanocarriers, developed by several pharmaceutical companies with a wide variety of formulations. In the preclinical study field, the use of human origin cells would also be ideal to avoid possible interspecies differences.

A highly reproducible human BBB in vitro model was developed and patented [20] by using CD34^+^ stem cell-derived endothelial cells (CD34^+^-ECs) seeded in the upper compartment of an insert and representing the luminal compartment (blood side) in a noncontact coculture during 6 days with brain pericytes seeded in the bottom wells, representing the brain compartment. In these culture conditions, the CD34^+^-ECs acquired BBB endothelial cells phenotype properties [17,21,22] and were named brain-like endothelial cells (BLECs). This model was developed using the 12-wells Transwell (TW) system and is routinely used to assess molecules passage across the human BBB but, also, to study the BBB either in pathological or physiological conditions. Indeed, this model showed, for the first time, a good correlation between the unbound fraction ratios brain/plasma-predicted in vitro, and the unbound fraction ratios CSF/plasma in humans [20].

Therefore, in order to better adapt to the current pharmacological development speed demand and to overcome this high yield need in early-stage drug discovery for compound screenings, inside the BBB field, we reported here the development of a miniaturized replicate from the already well-established and patented human BBB in vitro model for the HTS of compounds using automated technology. The characterization of this miniaturized and automated 96-well model was performed by comparison with the original manually established 12-well model. Molecule screening applications were tested for their toxicity, cell uptake and cerebral penetration rate.

## 2. Materials and Methods

### 2.1. Human BBB Models Setting Up

Human BBB in vitro models were produced using endothelial cells (ECs), derived from CD34^+^ hematopoietic stem cells and isolated from human umbilical cord blood according to the method described by Pedroso et al. [23]. The protocol was approved by the French Ministry of Higher Education and Research (reference: CODECOH DC2011-1321), and the sample collection was obtained under the written and informed consent from the donor’s parent of umbilical cord blood, in accordance with the French Legislation. Once isolated from umbilical cord blood, CD34^+^ cells were differentiated in vitro into ECs using endothelial cell growth medium (EGM; Lonza, Walkersville, MD, USA) containing 50-ng/mL vascular endothelial growth factor (PeproTech, Rocky Hill, CT, USA) and 20% fetal calf serum (FCS; Sigma Aldrich, Darmstadt, Germany). Then, brain-like endothelial cells (BLECs) were obtained using the Transwell system after noncontact cocultured with bovine brain pericytes (PCs) [24].

After two days of cell thawing and growing in petri dishes, noncontact cocultures were reproduced by firstly seeding bovine brain pericytes (PCs) on bottom-well plates coated with gelatine, using a basal endothelial cell medium (ECM; Sciencell, Carlsbad, CA, USA) supplemented with 5% FCS, 1% endothelial cell growth supplement (Sciencell, Carlsbad, CA, USA) and 0.5% gentamicin (Biochrom AG, Berlin, Germany), and placed for 3 h at 37 °C. After, CD34+-derived ECs were seeded on Transwell (TW) inserts coated with Matrigel™ (BD Biosciences, San Jose, CA, USA) diluted 1/48 (*v*/*v*) and immediately cocultured with the brain PCs. The cell medium was changed/refreshed two times before the models were ready after 6 days in the coculture. The same protocol was applied to develop both a BBB 12-well TW model and miniaturized 96-well TW model. At the end of 6 days in a coculture with PCs, the models were used in cocultures or in monocultures by transferring the inserts into new bottom wells without PCs seeded, depending on the approached experiment.

#### 2.1.1. Miniaturization

As a control, original human BBB models (12-well format) were always developed by seeding 80,000 ECs per filter, using 12-well TW plates (0.4-µm polycarbonate; ref. 3401; Corning^®^, New York, NY, USA), where the inserts had a growth surface area of 1.12 cm^2^ in a coculture with 50,000 PCs per bottom well.

Whereas a miniaturized model (96-well format) was developed either in two different 96-multiwell insert systems: Falcon plates (1-µm polyethylene terephthalate (PET); ref. 351130, Corning Life science, Tewksbury, MA, USA) with 0.0804 cm^2^ cell growth surface area and Corning plates (1-µm polyethylene terephthalate (PET); ref. 3380 Corning, New York, NY, USA) with 0.143 cm^2^ filter cell growth surface area. A test to find out the best cell seeding density ratio between brain pericytes (PCs) and CD34^+^ stem cell-derived endothelial cells (ECs) within the 96-multiwell insert systems was done by seeding different cell density ratios, starting from 8000 to 26,000 ECs in filters (luminal compartment) and from 6000 to 15,000 PCs per bottom well (abluminal compartment). Optimum cell seeding density ratio was assessed with the evaluation of the BBB integrity after 6 days of coculture by measuring the endothelial permeability coefficient (Pe) of the fluorescence paracellular marker sodium fluorescein (NaF), as detailed below in Section 2.2.2. Values were compared with the Original 12 TW model.

#### 2.1.2. Automation

##### Cell Seeding

Original BBB 12-well TW models were always seeded manually. However, for miniaturized 96-TW system models, after the manual cell seeding density ratio was obtained, a second test was done in order to confirm the optimal cell seeding density by using the cell seeding robot Multiflo FX (BioTek Instruments, Winooski, VT, USA). Then, the cell seeding in miniaturized models was routinely automated.

##### Immunochemistry and BBB Permeability Assays

Immunochemistry and permeability assays of integrity marker Sodium Fluorescein (NaF) and/or other compounds tested in the BBB miniaturized 96-multiwell insert system were completely automated using a robot Caliper (Sciclone (R), Perkin-Elmer, Waltham, MA, USA), by setting up the detailed protocols in the software, covering the different steps, volumes and working distances. In parallel, the immunochemistry and permeability assays in BBB 12-well TW models were performed manually.

##### Microscopy

Automated microscope ImageXpress Micro Confocal High-Content Imaging System (Molecular Devices, San José, CA, USA) was used for picture acquisition. Filters with ECs of 12-well TW models were cut and mounted on a glass slide under a coverslip, whereas ECs in filters of 96-multiwell insert systems were placed directly into a 3D frame previously developed and adapted for the direct filter visualization in the confocal microscope, removing the bottom plate. PCs of both systems were visualised directly in the bottoms of 12-well and 96-bottom well plates, respectively.

### 2.2. BBB Phenotype Validation of Miniaturized and Automated Human BBB In Vitro Model 

#### 2.2.1. Immunocytochemical Characterization

BLECs were fixed on their inserts using paraformaldehyde 10% *w*/*v* (PFA, ref HT5014; Sigma Aldrich, Darmstadt, Germany) during 10 min. Then, the cells were washed 3 times during 5 min with calcium and magnesium-free phosphate buffered saline (PBS-CMF). Membrane permeabilization was done with Triton 0.1% (Sigma-Aldrich, Darmstadt, Germany), and the cells were again washed 3 times during 5 min with PBS-CMF. Cells were incubated with SEA BLOCK blocking buffer (ref. 37527; Thermo Fisher Scientific, Waltham, MA, USA) for 30 min. After cell fixation, ECs were incubated in the dark for 1 h at room temperature with primary antibodies against zonula occludens-1 (ZO-1) (1/500; ref. 617300; Invitrogen; all from Thermo Fisher Scientific, Waltham, MA, USA), Claudin-5 (1/200; Polyclonal, Invitrogen™; Thermo Fisher Scientific), VE-Cadherin (1/500; Polyclonal, Invitrogen™; Thermo Fisher Scientific) and PECAM (CD31^+^) (1/50; Polyclonal, Invitrogen™; Thermo Fisher Scientific), which were diluted in PBS-CMF supplemented with 2% (*v*/*v*) normal goat serum (NGS). After three washing steps with 2%NGS-PBS-CMF, cells were incubated with secondary antibodies for 30 min at RT using goat antirabbit Alexa Fluor 568 (Invitrogen; dilution: 1/1000 in 2%NGS-PBS-CMF) for ECs. Nuclei were stained using Hoechst reagent (ref. B2883; Sigma Aldrich, Darmstadt, Germany) diluted by 1000 in the same secondary antibody solution. Finally, the cells were washed 3 times with PBS-CMF. 

Stained preparations were observed with the automated microscope ImageXpress Micro Confocal High-Content Imaging System (Molecular Devices, San Diego, CA, USA), using a blue DAPI filter for nuclei and green FITC filter for Z0-1, Claudin-5, Ve-Caherin and PECAM (CD31^+^). Images were processed using in MetaXpress software, version 6.5.2 (2018, Molecular Devices, LLC, San Jose, CA, USA).

#### 2.2.2. BBB Integrity Assay

BLEC physical integrity was evaluated by measuring the diffusion of sodium fluorescein (NaF; Sigma Aldrich, Darmstadt, Germany), a small hydrophilic molecule which poorly crosses the BBB. To do so, the ECs inserts were transferred into new that, 96-well plates (Falcon HTS 96-square well; ref. 353925; Corning Life Science, Tewksbury, MA, USA) or 12 well-plate depending on the model, containing 300 µL or 1.5 mL of Ringer-HEPES solution (150-mM NaCl, 5.2-mM KCl, 2.2-mM CaCl_2_, 0.2-mM MgCl_2_·6H_2_O, 6-mM NaHCO_3_, 5-mM HEPES and 2.8-mM glucose; pH 7.4) per well, respectively, which constituted the abluminal compartment. The cell culture medium from the EC inserts was removed, and 70 µL (96 TW system) or 500 µL (12 TW model) of Ringer-HEPES solution containing 1-µM NaF (the integrity marker) were added in the upper (luminal) compartment. The cells were incubated at 37 °C for 1 h. After the end of the incubation time, aliquots from the lower (200 µL) and upper (20 µL) compartments were collected in a 96-well plate for quantification, as well as aliquots from the initial solution at time zero (20 µL). Measurements were done with a fluorometer (Synergy H1; BioTek, Winooski, VT, USA) in a wavelength of absorption max. = 490 nm and emission max. = 525 nm. The endothelial permeability coefficient (Pe) of NaF was calculated in cm/min. The clearance principle was used to obtain the transport concentration-independent index. Briefly, the mean volume cleared was plotted against time, and the slope was estimated by linear regression. The permeability values of the inserts (PSf for inserts with a Matrigel™ coating only) and the inserts with ECs (PSt, Matrigel™-coated inserts with cells) were taken into consideration by applying the following equation: 

Equation (1):

1/PSe = 1/PSt − 1/PSf(1)

To obtain the BLEC permeability coefficient (Pe in cm/min), the permeability value (PSe) corresponding to the transport through the endothelium was divided by the insert’s membrane growth surface area (12 TW model, 1.13 cm^2^; 96 TW systems: Falcon, 0.0804 cm^2^; Corning, 0.143 cm^2^).

#### 2.2.3. RNA Extraction and Gene Expression Analysis

Gene profile was evaluated after 6 days of coculture for both models. Endothelial cells were rinsed 3 times with a warm RH buffer. BLECs were lysed from filters using RP1 buffer from the kit NucleoSpin^®^ RNA/protein from Macherey-Nagel (MACHEREY-NAGEL, Dueren, Germany). The mRNA was extracted, according to the manufacturer’s protocol. The purity and concentration were assessed measuring the absorbance at 260, 280 and 320 nm using a BioTek Synergy^TM^ H1 spectrophotometer and Take 3TM plate (BioTek Instruments, Winooski, VT, USA). For reverse transcription (RT), cDNA was obtained from 250 ng of mRNA using an IScript™ Reverse Transcription Supermix (Bio-Rad, Hercules, CA, USA). The qPCR reactions (10 µL) were prepared using a SsoFast™ EvaGreen^®^ Supermix (Bio-Rad) with water, cDNA, and primers at a final concentration of 400 nM. qPCR amplification was carried out in a CFX96 thermal cycler (Bio-Rad). Delta-Ct data was obtained from Bio-Rad CFX Manager software (Bio-Rad). Gene expression levels of the gene targets (Table 1) were calculated in base of the relative housekeeping gene RLP0 (ribosomal protein lateral stalk subunit P0) expression. 

#### 2.2.4. Efflux Pumps Functionality

After 6 days of coculture, the activity of the efflux transporters P-gp and BCRP in BLECs was assessed by performing the accumulation of Rhodamine 123 (R123). Hence, BLECs of the 96 TW systems and 12 TW model (as control) inserts were transferred in new well plates filled with 275–300 µL and 1.5 mL RH, respectively. The cell culture medium in the luminal compartments was removed, and ECs were incubated with 70 µL or 500 µL, respectively, of R123 (5 µM; Sigma Aldrich, Darmstadt, Germany) in RH with or without Elacridar (0.5 µM; Sigma Aldrich, Darmstadt, Germany). After 2 h at 37 °C, the reaction was stopped by rinsing the cells 5 times with ice-cold RH solution. ECs were lysed with RIPA buffer (Merck Millipore, Burlington, MA, USA). The quantification of R123 (excitation/emission wavelengths: 501/538 nm) in the samples was carried out using the fluorimeter Synergy™ H1 (BioTek Instruments, Winooski, VT, USA).

### 2.3. BBB Model Applications

#### 2.3.1. Drugs Rate Delivery to the Brain

A set of different compounds/drugs, Atenolol, Bupropion, Diazepam, Indomethacin, Lamotrigine, Levofloxacin, Methotrexate, Metoprolol and Verapamil (from Sigma Aldrich, Darmstadt, Germany), were tested. Prior, pericytes and BLECs were rinsed 3 times with a warm RH solution. Abluminal (receiver) compartments were filled with 300 µL of RH. In the luminal (donor) compartment, 70 µL of tested drugs at 2 µM in RH solution with 0.5% human serum albumin were added. After 3 h of incubation, aliquots from the donor and receiver compartments were taken and analyzed using a LC–MS/MS system consisting of an AB SCIEX TripleTOF^®^ 5600 mass spectrometer (AB Sciex, Singapore) outfitted with an Eksigent Ekspert™ nano LC 400 System. The free fraction ratios of the brain/plasma in vitro (Kp,uu,brain) were generated using the free drug concentration in the receiver compartment and in the donor compartment, following Equations (2) and (3). Results were compared to unbound fraction ratios of CSF to plasma (Kp,uu,CSF) in humans taken from the dataset already published [25].

Equation (2):

In vitro Kp,uu,brain = (C0 − CD)/CD) × U(2)

Equation (3):

% Recovery (U) = (CD + CR) × 100)/C0(3)

where C0 is the concentration of the molecule in the donor compartment at time zero, CD and CR are the free drug concentration at 3 h in the donor compartment (luminal side) and in the receiver compartment (abluminal side), respectively. U is the percentage of the recovered amount of the unbound molecule total in the donor and receiver compartments at 3 h.

#### 2.3.2. Compounds BBB Impact

A set of different neural impact compounds, L-ascorbic acid, acetaminophen, troglitazone, tamoxifen and rotenone, were incubated during 24 h diluted in a cell culture medium (ECM5) in the luminal compartment of the 96 TW system through eleven different concentrations following a dilutions series (0.001, 0.003, 0.01, 0.03, 0.1, 0.3, 1, 3, 10, 30 and 100 µM). After the incubation time was achieved, the BBB integrity of the model was evaluated by measuring the endothelial permeability coefficient of the integrity marker NaF (Pe^NaF^ cm/min), as described in the section above. 

#### 2.3.3. Cell Uptake and Endocytic Route Inhibitors 

The receptor functionality by endocytic cell uptake of the target molecule acetylated LDL fluorescence-labeled Alexa Fluor ™ 488 (488 acLDL; ref: L23380, Invitrogen™, Waltham, MA, USA), was assessed by molecule incubation within the BLECs and by using a set of inhibitors for active transport (carbonyl cyanide 4-(trifluoromethoxy) phenylhydrazone) FCCP; 20 μg/mL; ref. ab120081; Abcam, Cambridge, MA, USA) for the clathrin-mediated transcytosis (Genistein; 15 μg/mL; ref. G6649; Sigma-Aldrich, Merck KGaA Darmstadt, Germany), caveolae-mediated transcytosis (Chlorpromazine; 20 μg/mL; ref. C8138; Sigma-Aldrich, Merck KGaA Darmstadt, Germany) and macropinocytosis (Dimethylamiloride; 20 μM; ref. A4562; Sigma-Aldrich, Merck KGaA, Darmstadt, Germany) pathways. The BBB impact of those inhibitor concentrations were evaluated prior. Hence, the inhibitors were preincubated for 45 min and diluted in RH supplemented with 0.1% BSA; after, acLDL was added and incubated during 1 h at 15 μg/mL. AcLDL, used as the uptake control, was incubated during 1 h without any inhibitor in the medium. At the end of the experiment, BLECs were rinsed 5 times with cold RH. Cells were fixed with PFA 10%. Nuclei were stained using Hoechst reagent. Samples were observed with the automated microscope, using a blue DAPI filter for nuclei and green FITC filter for acLDL. Images were processed in MetaXpress 6.5.2 (2018, Molecular Devices, LLC, San José, CA, USA).

### 2.4. Statistical Analysis 

Results were expressed as the mean ± SD or SEM. The number of replicates (*n*), performed through independent experiments (N), were detailed in each experiment. For the analysis involving different groups, an unpaired *t*-test ormultiple comparison tests (ordinary one-way ANOVA) were used or multiple *t*-test when there were different conditions between groups. For correlation, a nonlinear regression analysis was used. The thresholds for statistical significance were set to * *p*-value < 0.05 and ** *p*-value < 0.01. All data was analyzed by the GraphPad Prism 8.1 software (GraphPad Software, San José, CA, USA).

## 3. Results

### 3.1. Miniaturization and Automation of a Human In Vitro BBB Model

#### 3.1.1. Cell Culture, Plates and Volumes 

**Figure 1:**
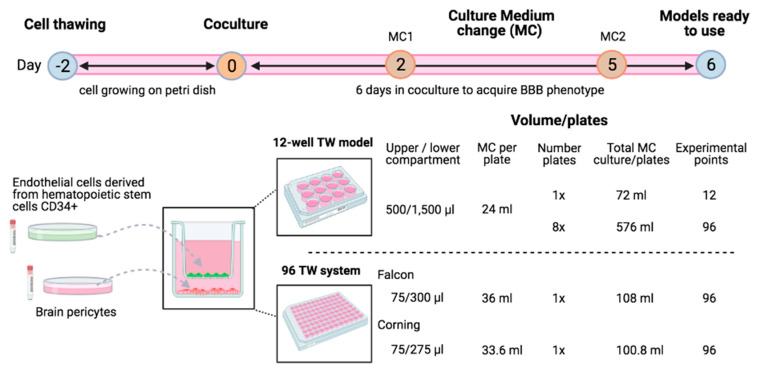
Schematic representation of the cell culture process to develop the noncontact coculture humanin vitroBBB models using endothelial cells (ECs) derived from human hematopoïetic stem cells and bovine brain pericytes. Cells were thawed in petri dishes and let grown for 2 days. Then, cocultures were settled by seeding human ECs in the filters and brain pericytes in the bottom compartment either for 12-well plates or miniaturized 96 TW systems. After 2 media changes (MC), experiments were then realized on day 6 of the coculture needed to acquire the BBB phenotype. Volume of the culture medium used regarding the type and number of plates was detailed. Image created with BioRender.com (accessed on 15 April 2021).

Both (12-well Transwell plates (TW) and 96 TW) models were developed following the same cell culture protocols concerning the time, steps and components. Endothelial cells derived from hematopoietic stem cells CD34^+^ and brain pericytes were thawed and let grown in petri dishes two days before the cells seeding in the TW, counting this step as day 0. Then, 6 days of cell coculture were required for the BBB model differentiation, including two cell medium changes (Figure 1). On day 6, the models were ready to be used for their phenotypic and functional evaluation. 

Volume of material needed deferred for each system: To develop the original 12-well TW BBB model, the volume of gelatine required to coat bottom wells for pericyte seeding was 1000 µL per well and 500 µL for Matrigel™ coating per filter to seed the ECs (i.e., 6 mL of Matrigel™ for 12 experimental points). Moreover, the cell culture medium (ECM5) in the upper and lower compartments were 500 and 1500 µL, respectively, meaning a 24-mL medium used per full plate in a total of 72 mL per 12 experimental points developed, including the renewal medium needed during the 6 days of culture. Then, concerning the miniaturized human BBB model replicates, the volume required for gelatine coating was 75 µL per bottom well and 35 µL Matrigel™ coating per filter (i.e., 3.4 mL of Matrigel™ for 96 experimental points). The cell culture medium requirement was75 and 275–300 µL in the compartments; in a total of 33–36 mL per full plate, consequently, 100–108 mL for 6 days of culture were needed to develop 96 experimental points.

Hence, by the same cell culture time invested, miniaturized models allowed the much-increased amount of experimental points. In addition, the quantity of the material and plastic waste were inversely proportional to the number of experimental points needed. Indeed, eight plates of 12-well TW would be needed to achieve 96 experimental points, meaning 576-mL ECM, while the miniaturized system required just 108-mL ECM for one plate 96 TW, saving seven plastic waste plates compared to in the original conditions. Therefore, concerning the material and plastic wastes, miniaturized systems have started being much more profitable when more than 12 experimental points were needed, which involved the investment of more than one 12-well TW plate.

#### 3.1.2. Adaptation of the Cell Densities to the Miniaturization 

**Figure 2:**
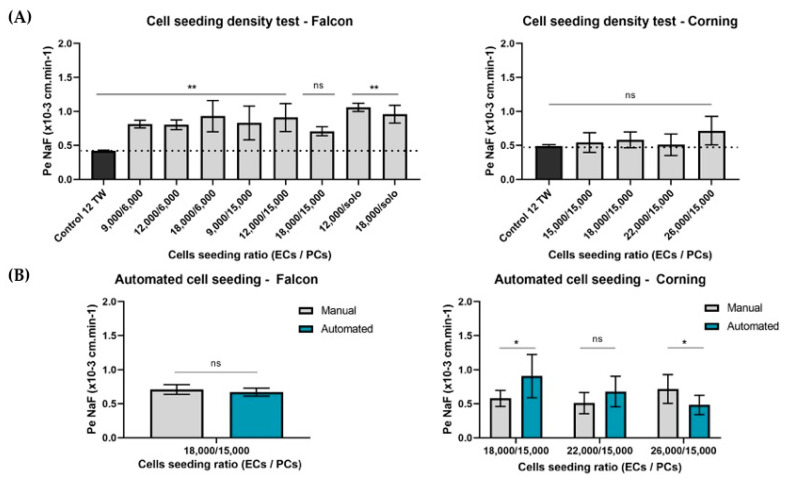
Adaptation of the cell seeding densities for the miniaturized BBB model development. Endothelial cells (ECs) were seeded on the filters of the insert system and pericytes (PCs) in the bottom wells in different densities. (**A**)The manual cell seeding density ratio test for the different 96-multiwell system plates by a comparison of the Pe^NaF^ coefficients was used as the integrity marker for the model, obtained from the 12 TW BBB model used as the control (*n* = 3 replicates) and the miniaturized systems. Statistical analyses were done using ordinary one-way ANOVA with multiple comparison tests. Significant differences were ** *p*-value < 0.01, and ns = no significant differences. Hand cell seeding density ratio test in Falcon plates, *n* = 8 replicates; Corning plates, *n* = 6 replicates. (**B**)Validation of the cell seeding automation test. Falcon plates automated cell seeded by the robot showed no significant differences (ns) in the EC integrity compared with hand seeding. Corning plates showed significant differences according to the cell seeding type: 18,000/15,000 and 26,000/15,000 (ECs/PCs), * *p*-value < 0.05, and no significant differences (ns, *p* = 0.109) for the automated cell seeding ratio 22,000/15,000 (ECs/PCs), *n* = 8 replicates. Statistical analyses were a multiple *t*-test.

The first step of the miniaturization was achieved by adapting manually the cell seeding density ratio. After 6 days of coculture, tightness of the BLEC monolayer in the miniaturized model was measured, comparing the endothelial paracellular permeability coefficient to the BBB integrity marker sodium fluorescein (Pe^NaF^) with the value obtained in the original 12-well model as the control (Figure 2). Firstly, the analysis for 96 TW Falcon^®^ plates, which presented a filter cell growth area of 0.0804 cm^2^, the endothelial cells (ECs) density seeded in the filters were 9000, 12,000 and 18,000 ECs per filter in a coculture with the brain pericytes (PCs), where the densities analyzed were in 6000 and 15,000 PCs per bottom well, as well as a condition of a monoculture (solo) with no presence of pericytes. The results showed that, for the Falcon^®^ 96 multiwell insert system, the cell ratio of ECs/PCs that did not present significant differences (ns, *p* = 0.1864) with the control 12 TW model was found in the density ratio 18,000/15,000 (ECs/PCs) per insert/well (Figure 2A). However, for the Corning^®^ plates, which presented a filter cell growth area of 0.143 cm^2^, the cell density of the ECs analyzed were increased in-line with the increased area. Hence, 15,000, 18,000, 22,000 and 26,000 ECs per filter were tested in a coculture with the best PC density obtained previously of 15,000 PCs per bottom wells. The results showed a much-achieved low Pe^NaF^ coefficient with no significant results (*p* > 0.05) compared with the control of the original 12-well TW model.

#### 3.1.3. Automation

##### Cell Seeding Densities

After the manual cell seeding density was evaluated, a second cell density ratio test was assessed to validate its suitability by using an automated cell seeding machine Multiflo (BioTek Instruments, Winooski, VT, USA). For Falcon plates, the best manual seeding density was found in 18,000/15,000 (ECs/PCs) per insert/well tested using the robot. The results obtained showed no significant differences (*p* = 0.240) between the hand and automated cell seeding in Falcon^®^ plates. However, for Corning plates, the number of ECs seeded must be increased up to 22,000 ECs/filter (Figure 2B) to maintain a BBB tightly packed network checked by the low permeability coefficient to integrity marker NaF.

##### Cell Visualization

**Figure 3:**
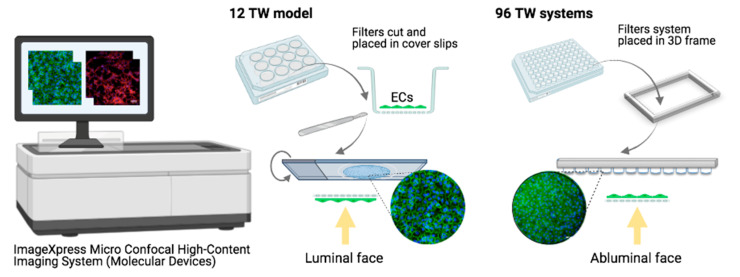
Schematic representation of endothelial cell visualization by confocal microscopy. In the original 12 TW model, filters were cut and placed in coverslips, then returned to acquire the pictures over the luminal faces of the ECs. Where filters from the miniaturized systems were directly placed on a 3D frame developed and adapted to the microscope, avoiding the need of the bottom compartment, reducing the lens height of going through the two compartments, pictures were taken from the abluminal EC face by going through the filter. Image created with BioRender.com (accessed on 15 April 2021).

Automation was also achieved for cell visualization, using the ImageXpress Micro Confocal High-Content Imaging System (Molecular Devices, San Diego, CA, USA). On the one hand, to visualize the endothelial cells seeded filters of the 12-well TW model, the filters were cut and placed one by one into coverslips, which made the process longer and handwork more dedicated. However, in that case, pictures were acquired from the ECs luminal face, since this format allowed the possibility to turn the coverslip; hence, pictures acquired clearer and sharper images. On the other hand, the miniaturized 96 TW model was completely automated by directly placing the filters on a 3D frame specially created based on the plate’s measurements and adapted for the Micro Confocal High-Content Imaging System. Hence, no previous manual work was required, and the time was highly decreased. The use of the so-called adapted 3D frame allowed the possibility of a direct visualization to the cells in the filters by removing the bottom compartment, which highly interfered in the quality of the images. However, despite the high improvement in the image quality, the cells were still always visualized by the abluminal face, with the light having to cross the insert membrane (Figure 3). Then, in both models, the bottom wells where the PCs were seeded were directly placed, and visualization was automated. However, PCs visualization was not possible in the Falcon plates because of the angled design of the bottom well not adapted being for cell visualization.

##### Experimental Assays

After succeeding in the miniaturization by finding the optimum cell seeding density ratio for each plate, followed by the automated cell seeding procedure and cell visualization protocol adapted in the base of each system characteristic, the BBB permeability assays for the screening compounds and/or BBB integrity test using the marker NaF, as well as immunostaining assays, were also automated by using Caliper (Sciclone(R), Perkin-Elmer, USA) for the miniaturized BBB model by setting up the protocol followed, while the permeability and immunochemistry experiments in the 12-well TW model were done by hand (Table 2).

### 3.2. BBB Phenotype Maintenance of Miniaturized and Automated Human BBB In Vitro Model Replicate

#### 3.2.1. BBB Gene Profile Maintenance

**Figure 4:**
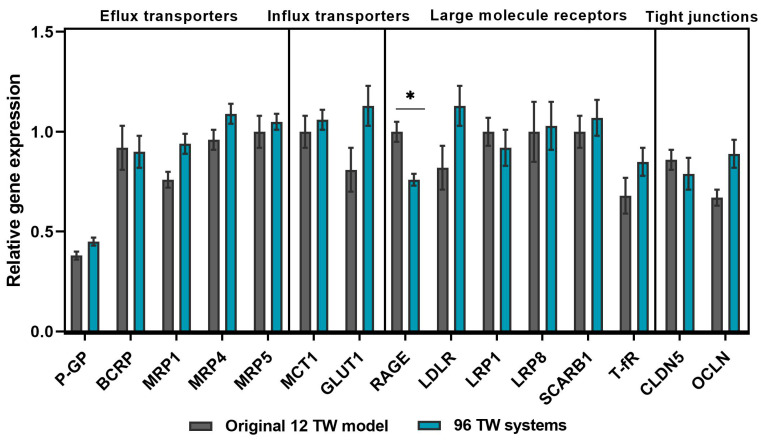
Gene expression analysis by RT-qPCR. Profile study comparison between the original 12 TW model (grey bars) and miniaturized 96 TW system (blue bars) of the efflux and influx transporters, large-molecule receptors and tight junctions. Samples were *n* = 9 in *N* = 3 (except for LRP1: *n* = 6 and *N* = 2). Relative normalized expression with RPLP0. Mean ± SEM. Multiple *t*-test showed no significant differences for: P-gP (*p* = 0.261), BCRP (*p* = 0.988), MRP1 (*p* = 0.126), MRP4 (*p* = 0.548), MRP5 (*p* = 0.988), MCT1 (*p* = 0.988), GLUT1 (*p* = 0.410), LDLR (*p* = 0.422), T-fR (*p* = 0.988), LRP1 (*p* = 0.988), LRP8 (*p* = 0.988), SCARB1 (*p* = 0.732), CLDN5 (*p* = 0.988) and OCLN (*p* = 0.176), except for RAGE gene expression (* *p* = 0.0120).

In order to acquire a varied range of gene profile study in both system, different genes were targeted to evaluate their expression: efflux transporters (P-gP, BCRP, MRP1, MRP4 and MRP5); influx transporters (MCT1 and GLUT1); large molecule receptors (RAGE, LDLR, T-fR, LRP1, LRP8 and SCARB1) and tight junctions (CLDN5 and OCLN) (Figure 4). The results showed a stable profile maintained in both models, with no significant differences (*p* > 0.05), except for RAGE gene expression (*p* = 0.0120), which differed significantly between both models. The results were normalized with the housekeeping gene RLP0.

#### 3.2.2. Low Paracellular Permeability of a Tightly Packed BLECs Network

**Figure 5:**
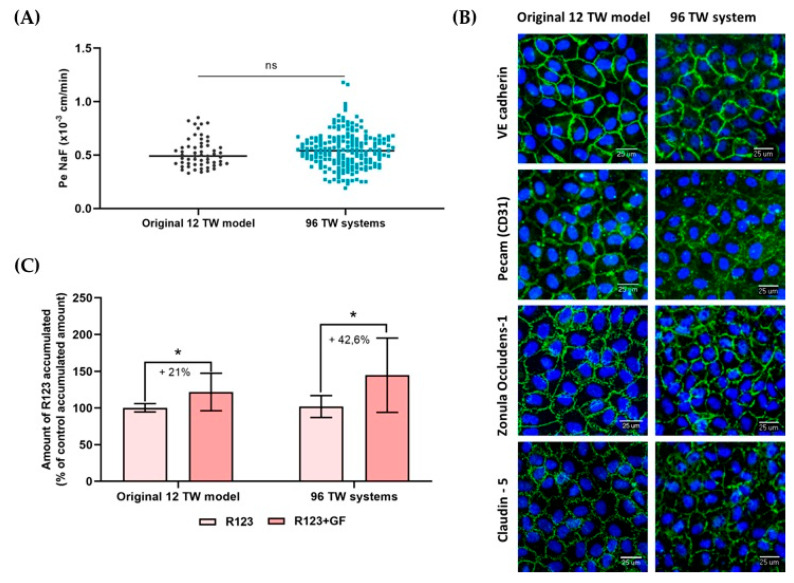
BBB phenotype comparison between the original 12 TW model and the miniaturized 96 TW system. (**A**) Endothelial permeability coefficient (Pe in cm/min) measuring the transport of sodium fluorescein (NaF) through the BLECs in the original 12 TW model and the miniaturized 96 TW system over the different cell seeding series. The results are presented in a scatter plot for the original 12 TW models: *n* = 57, *N* = 19 and 96 TW systems: *n* = 194, *N* = 22 (unpaired *t*-test; ns = no significant differences). (**B**) Immunocytochemical CD34 ^+^-ECs BBB phenotype validated by the expression of the proteins involved in the junctions (in green): VE cadherin, PECAM (CD31^+^), Zonula occludens-1 and Claudin-5. Nuclei are in blue. Original 12 TW images were taken from the luminal BLEC face, while images in the 96 TW system were taken from the abluminal BLEC face, using the ImageXpress Micro Confocal High-Content Imaging System, objective 20× (scale bar = 25 µm). (**C**) Assessment of the efflux pump functionality. Percentage of the R123 intracellular accumulation in the BLECs in the presence or absence of the inhibitor Elacridar (GF) in the original model and in the miniaturized model. Samples: *n* = 9, *N* = 3 (multiple *t*-test; * *p*-value < 0.05).

Throughout the different cell seeding series developed for the experiments described herein using both the 96 TW systems, as well as for the original 12 TW model developed, the endothelial cell permeability coefficient (Pe) of the fluorescent BBB integrity marker sodium fluorescein (NaF; 1µM) was evaluated. The value of this coefficient gave a feedback of the BBB integrity of the model. The results showed that Pe^NaF^ presented no significant differences between the original 12 TW model and 96 TW model (unpaired *t*-test; ns = no significant differences, *p* = 0.2308), which remained low in both models (Figure 5A), suggesting a restrictive paracellular pathway, which was supported by the immunocytochemical assays showing that BLECs formed a regular monolayer with a tightly packed network and non overlapping cells with the presence of characteristic adherens junction proteins (VE Cadherin and PECAM) and tight junction proteins (Zonula Occludens-1 and Claudin-5) located at the cell edges (Figure 5B).

#### 3.2.3. Efflux Pumps Functionality

Functionality of the efflux pumps (P-gp, BCRP) were evidenced by assessing the intracellular accumulation of the substrate rhodamine123 (R123) within the BLECs (Figure 5C) in the presence of the efflux pump inhibitor Elacridar (GF). The results showed that significant differences (multiple *t*-test, * *p*-value < 0.05) in the R123 intracellular accumulation increased in the GF inhibitor presence compared with the control. The original 12 TW model presented an intracellular accumulation of 21%, and in the miniaturized 96 TW system, this accumulation achieved 42.6%.

Therefore, those first results showed that the endothelial cells of the miniaturized model after 6 days of a coculture presented a BBB phenotype, as expected in the original 12-well model used as the control.

### 3.3. Some Applications for Which the Miniaturized and Automated Human BBB In Vitro Model Have Been Successful

#### 3.3.1. Brain Exposure to the Drug Correlations with Human In Vivo Data

**Figure 6:**
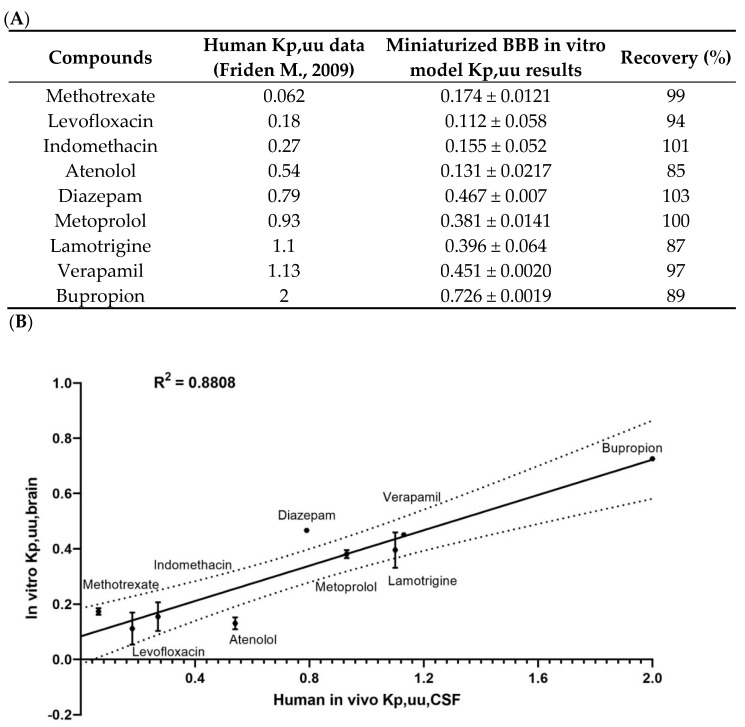
Screening application of the miniaturized and automated human BBB in vitro model. (**A**) Values (mean ± SD) of the unbound fraction ratio of the CSF/plasma in humans (in vivo Kp,uu,CSF) data obtained from Friden M et al. 2009. Unbound fraction ratios of the brain/plasma predicted in vitro (in vitro Kp,uu,brain) through the 96 TW miniaturized and automated human models. The concentrations were detected by mass spectrometry (LC–MS/MS system) with the calculation of the total amount of unbound molecules recovered (recovery, expressed in percentage). (**B**) Correlation between the Kp,uu,CSF and Kp,uu,brain nonlinear equations; R^2^ = 0.8808; *n* = 3; *N* = 1.

The screening of a set of compounds whose values for the free fraction ratio of the brain/plasma that are known in humans in vivo (Atenolol, Bupropion, Diazepam, Indomethacin, Lamotrigine, Levofloxacin, Methotrexate, Metoprolol and Verapamil) was assessed in the 96 TW system BBB model. The results obtained in vitro of the predicted unbound fraction ratio (in vitro Kp,uu,brain) were compared with the in vivo unbound fraction ratio in the cerebrospinal fluid (CSF) and plasma reported in humans [25] (in vivo Kp,uu,CSF) (Figure 6A). The results were plotted in a XY correlation, presenting the nonlinear regression with a value R^2^ = 0.8808 (Figure 6B), meaning a fair correlation between the data obtained in our miniaturized human in vitro BBB model and the data obtained in humans. Such a correlation (R^2^ = 0.84) was previously obtained using the 12-well human BBB in vitro model [20].

#### 3.3.2. Compound Impact Studies over the BBB 

**Figure 7:**
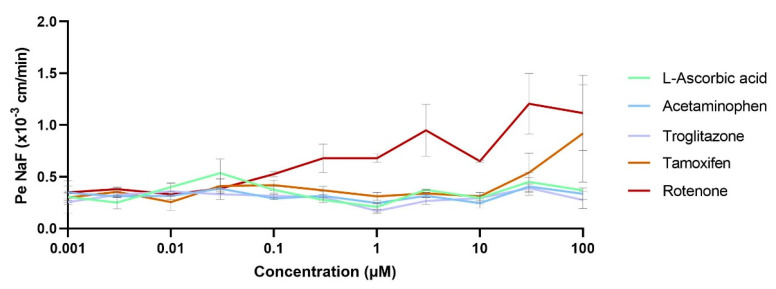
Compound impacts of the BBB through concentrations. A set of nontoxic (L-ascorbic acid), toxic compounds but non-neurotoxic (acetaminophen and troglitazone) and neurotoxic compounds (tamoxifen and rotenone) were incubated for24 h in the coculture medium; permeability coefficients for the BBB integrity marker NaF (Pe^NaF^) were assessed. Samples: *n* = 2, *N* = 2.

The BBB impacts on different toxic levels of compounds through a wide range of concentrations were evaluated using a set of nontoxic compounds (L-ascorbic acid), toxic compounds but non-neurotoxic (acetaminophen and troglitazone) and neurotoxic compounds (tamoxifen and rotenone). After 24-h incubation of the cocultures with the compound tested at different concentrations, the permeability coefficients of the BBB integrity marker NaF (Pe^NaF^) were calculated. The results showed that the BBB integrity was maintained around 0.33 ± 0.08 × 10^−3^ cm/min for the non-neurotoxic compounds L-ascorbic acid, acetaminophen and troglitazone (Figure 7). However, the neurotoxic compound rotenone started becoming toxic for the BBB in concentrations above 0.3 µM, showing a high BBB disruption at the maximum concentration tested of 100 µM (unpaired *t*-test, ** *p* = 0.0049). Moreover, the BBB showed affection in the presence of tamoxifen in concentrations above 30 µM without being highly disrupted at the maximum concentration tested of 100 µM (unpaired *t*-test, *p* = 0.328). 

#### 3.3.3. Molecular Internalization and Endocytic Routes Study

**Figure 8:**
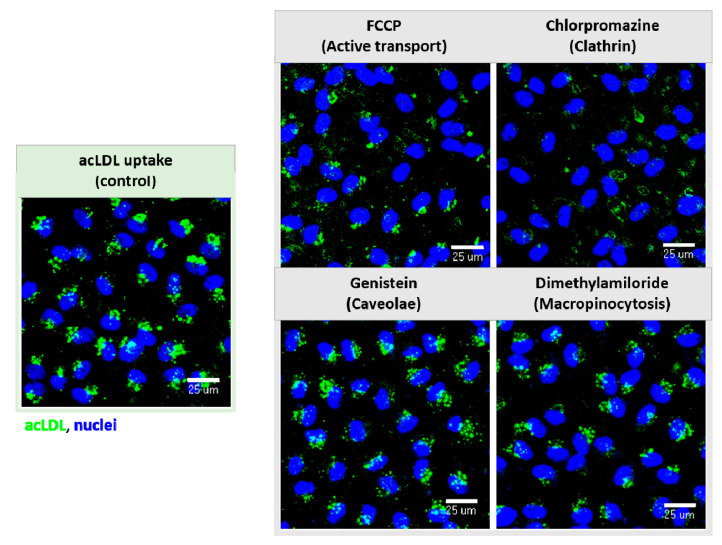
Receptor functionality and endocytic pathway inhibition. Fluorescence-labeled acetylated LDLs (acLDLs) exposed to the inhibitors for the ATP synthesis (FCCP), for caveolae (Genistein) and clathrin-mediated transcytosis (Chlorpromazine) and macropinocytosis (Dimethylamiloride) pathways. AcLDL uptake within the BLECs decreased when FCCP and Chlorpromazine were present, showing the functionality of the receptors. Images taken by the ImageXpress Micro Confocal High-Content Imaging System, objective 20× (scale bar = 25 µm).

As an example of the receptor functionality of the miniaturized model, the endocytic pathway of a well-studied molecule, acetylated low-density lipoproteins (acLDLs), which is known to interact with the scavenger receptor type B member I (SCARB-I) to be actively internalized within BLECs were chosen. AcLDL fluorescence labeled to be easily followed by microscopy was used. The pathway was assessed using inhibitors for ATP production (FCCP), caveolae-mediated endocytosis (Genistein), clathrin-mediated endocytosis (Chlorpromazine) and macropinocytosis (Dimethylamiloride). No BBB impact of the inhibitors at the used concentrations was previously demonstrated (data not shown). The results showed that acLDL (uptake control) was able to internalize within the BLECs. Then, the acLDL uptake was visibly reduced when the inhibitors for ATP synthesis (FCCP) and for clathrin-mediated endocytosis (Chlorpromazine) were present in the media (Figure 8) in comparison with the uptake control following the same incubation time, thus demonstrating that this process requires energy and is mediated by a receptor clathrin-mediated endocytosis. Genistein and Dimethylamiloride did not interfere with the acLDL uptake.

## 4. Discussion

A miniaturized design decreases the working surface area and, consequently, reduces the volume of compounds needed for its development and provides a highly increased amount of experimental points in the same cell culture time spent. This allows increasing the number of compounds to screen for or conditions to analyze within the brain cells (Figure 1). In addition, automation enables a robotized precision, researchers’ physical efforts and a time decrease. As an example, in this article, for the automated cell visualization of the 96 TW systems, a novel improved protocol was developed from the 3D frame strategy (Figure 3), where, by directly placing the inserts in the adapted frame for the microscope, allowed the achievement of more consistent quality cell images than those expected by the product manufacturer [26]. Since the bottom compartment to hold the insert was removed, the plastic layers and working lens distances were reduced. Hence, this miniaturized and automated replicate from the well-established and patented human BBB in vitro model [20] was developed with the aim to facilitate and speedup CNS drugs/compounds screening and/or research in early-stage drug discovery, either for pharmaceutical industries or academic research.

In the human brain, at the capillary level, endothelial cells express thousands of transcripts encoding different transporters, receptors, efflux pumps, ion channels, regulatory molecules and tight junction proteins. Some of them have the function to prevent the brain from accessing and accumulating drug conjugates and xenobiotics via an active efflux or specific transporters from the endothelium to the blood [3,27]. Hence, a gene profile study for the genes targeted for influx and efflux transporters, as well as the large molecule receptors and tight junction elements selected (Figure 4), showed their presence and stable maintenance of the gene expression between both formats. Then, this miniaturized and automated replicate also met the reliability criteria expected for an in vitro BBB model by presenting the specific properties of a human adult brain endothelial cell line [17,28,29], i.e., a confirmed and reproducible low-paracellular permeability, demonstrated by measuring the permeability coefficient (Pe) of sodium fluorescein (NaF), which is a small hydrophilic molecule that poorly crosses the BBB and was used as the integrity marker for our model (Figure 5A). Indeed, this coefficient value gave a feedback of the BBB integrity of the model for each cell seeding series, and it was used as the control to evaluate the impact of a compound over the BBB after its incubation. Moreover, it brought a value that could be easily used for the comparison and extrapolation between different BBB models due to its method of calculation, taking into account the parameters such as the filter surface area and volume ratio between compartments, as it was applied to compare the miniaturized and automated model herein presented with the original 12 TW format. The low-paracellular permeability was confirmed with the presence of a regular nonoverlapping monolayer with tightly packed cell networking (Figure 5B). In addition, efflux pump functionality was evidenced by assessing the intracellular accumulation of the substrate rhodamine123 within the BLECs (Figure 5C) in the presence of the efflux pump inhibitor Elacridar [17,30].

Thus, once the miniaturized and automated human in vitro BBB model was successfully accomplished, different examples of the applications of compounds to study were realized. Depending on the objective and the molecule studied, over the decades, different methods of experimental display and calculations have been described for the use of static in vitro BBB models [31]. As an example, the ratio between the unbound concentrations in the brain and plasma has been accepted as a major pharmacokinetic parameter in drug discovery; additionally, in vitro studies have been adapted to assess its predictions [32,33,34]. Hence, for this miniaturized BBB system, as it was previously demonstrated with the original model [20], the correlation between the unbound fractions ratio of the brain/plasma predicted in vitro from our miniaturized model and the in vivo unbound fractions ratio in the cerebrospinal fluid (CSF) and plasma reported in humans [25] again showed a high correlation (R^2^ = 0.8808) (Figure 6), which gave a good sign for the predictability values in the humans using the miniaturized and automated BBB in vitro model.

In addition, concerning the usability and principal applications for such in vitro models, the toxicity evaluation of the drugs/compounds is one of the main research points in early drug discovery, where many conditions related to a wide range of concentrations and incubation times must be deeply evaluated. In our study, the impact towards the BBB of different compounds, non-neurotoxic [35,36,37] and neurotoxic [38,39], through a wide range of concentrations was assessed. The permeability coefficient of our integrity marker NaF for the endothelium (Pe^NaF^) (Figure 7) showed an effect after exposure to high concentrations of neurotoxic compounds such as tamoxifen and rotenone, which were known to disrupt the BBB [38,39]. This miniaturized format allowed a much faster and easier evaluation of many conditions at one single time, which is of interest in neurotoxicity testing to study the impacts of increasing concentrations of compounds [40].

In the early-stage compounds study, there was also a great interest in the deep molecular research evaluation concerning the mechanisms of molecule transport within the cell or nanovector interactions with the cells [41]. Hence, many strategies such as the fluorescence labeling of the molecules [11] allows an easy follow-up in their transport by microscopy to evaluate their cellular uptake, as well as the deciphering of the mechanisms of internalization/transport, using surface inhibitors for the different internalization pathways. Then, as a probe of functionality and concept, a fluorescence-labeled molecule (acetylated-LDL) was used in our experiments. Acetylated-LDL is known to interact with the scavenger receptor, easily internalizing the brain ECs following a clathrin-mediated endocytosis to be degraded by the lysosomes [42,43]. In our study, the use of different pathways inhibitors [44,45,46], with a presented visible reduction in the molecular uptake (Figure 8), allowed us to show the receptor functionality of the endothelial cells and the feasibility of such experiments in a 96-well format. The same principle is applied to new molecules in research. Therefore, because of the wide range of possible studies involved either through a CNS set of drugs library [11,47,48] or a single selected molecule during early drug discovery, this miniaturized system allows a much faster HTS of the different compounds and experimental conditions.

However, despite this progress in the BBB in vitro models field, there are still several parameters that statics automated models cannot overcome, such as the microfluidic devices, with steering, and closed environment [19], in addition to the possibility of more than two brain cell type presences. Nevertheless, on this last point, the herein described miniaturized model could focus future perspectives on the addition of different brain cell types (astrocytes and neurons) in the bottom compartment of the model, as previously done with the 12 TW format [49]. Indeed, the use pericytes conditioned media to substitute the presence of the brain pericytes in the lower compartment [50], could help to induce a correct BBB phenotype of the BLECs, because of the small volume format per well, allowing the possibility of different cell types to be seeded in the abluminal compartment of the model. This configuration would give the supplementary possibility of a drug effects screening study of different brain cell types, such as neurons, astrocytes and glia, after transport across the BBB, possibly called “CNS drug effects of brain cell screening”.

## 5. Conclusions

Herein, for the first time in the field, the development of a miniaturized and automated in vitro human BBB model for the HTS of the compounds in 96-multiwell systems has increased the facility of CNS drug/compound screenings. This replicate met the expected criteria of an in vitro BBB model showing specific properties of the human adult brain endothelium by reproducing the in vivo BBB phenotype with low paracellular permeability and the presence of tight junctions, as well as efflux pumps and membrane receptor functionality and, most importantly, presenting a high correlation with human in vivo data concerning the brain exposure to drugs. Determining the free fractions ratio of the brain/plasma at an early stage in the drug discovery process helps to predict whether drug candidates are likely to achieve high-enough CNS exposure to elicit the desired pharmacological effect.

The system is easy to handle and uses automated technology, which, in the same cell culture time spent compared to the original 12 TW model, much data is obtained afterwards, offering the opportunity to generate useful numerous BBB permeation data of many CNS compounds at a time, consequently achieving a much faster screening or single-molecule research study while allowing a relevant reduction in the materials and plastic wastes, contributing to the footprint reduction of scientific research and development. Hence, this model appears to be more suitable than the previously characterized model for its use in HTS, which benefits its extensive use during early-stage drug discovery studies by either small or big pharmaceutical companies or academic research studies.

## Figures and Tables

**Table 1:** *Homo sapiens* DNA primers used to amplify the mRNA expression of: efflux transporters such as P-glycoprotein (P-gp), breast cancer resistance protein (BCRP) and multidrug resistance proteins (MRPs; subfamily of the ATP-binding cassette (ABC) transporters); influx transporters such as Monocarboxylate transporter 1 (MCT1) and Glucose transporter 1 (GLUT1); large molecule receptors like the receptor for advanced glycation end products (RAGE), low-density lipoprotein receptor (LDLR) and related proteins (LRP1 and LRP8), Scavenger receptor class B type 1 (SCARB1) and transferrin receptor (T-RF), as well as junctional proteins Claudin-5 (CLDN5) and Occludin (OCLN): in the BLEC monolayer from the miniaturized and original in vitro BBB models.

Target	Gene	AN *	Primer F/R	Primer Sequence
P-GP	ABCB1	NM_001348945	F	CAGACAGCAGCTGACAGTCCAAGAACAGGACT
			R	GCCTGGCAGCTGGAAGACAAATACACAAAATT
BCRP	ABCG2	NM_001348989	F	TGGCTGTCATGGCTTGAGTA
			R	GCCACGTGATTCTTCCACAA
MRP1	ABCC1	NM_004996	F	GTCCTTAAACAAGGAGGACACG
			R	TCCTTGGAGGAGTACACAACCT
MRP4	ABCC4	NM_005845	F	ACCTTGCAAGAGCAGTGTATCA
			R	TGTCTGCTAACTTCCGCATCTA
MRP5	ABCC5	NR_135125	F	CCCAGTCCTGGGTATAGAAGTG
			R	CGAGTTCTCCTGAACTTGGAAT
MCT1	SLC16A1	NM_001166496	F	AAGGTATATTCCATGCCACCAC
			R	GCTGATAGGACCTCCACCATAC
GLUT1	SLC2A1	NM_006516	F	CTTCTCCAACTGGACCTCAAAT
			R	AGGAGCACAGTGAAGATGATGA
RAGE	AGER	NM_001206929	F	GAGTCCGTGTCTACCAGATTCC
			R	ATCCAAGTGCCAGCTAAGAGTC
LDLR	LDLR	NM_000527	F	TTCATGGCTTCATGTACTGGAC
			R	TTTTCAGTCACCAGCGAGTAGA
LRP1	LRP1	NM_002332	F	AATGAGTGTCTCAGCCGCAA
			R	AACGGTTCCTCGTCAGTCAC
LRP8	LRP8	NM_033300	F	TGTTTTGCATAATCCAGCAATC
			R	GGTCAACTGCATTTACCCTCTC
SCARB1	SCARB1	NR_160416	F	ATCCCCTTCTATCTCTCCGTCT
			R	GTCGTTGTTGTTGAAGGTGATG
T-FR	T-fR	NM_001313965	F	ACTTCTTCCGTGCTACTTCCA
			R	CCACTCTCATGACACGATCATT
CLDN5	CLDN5	NM_003277	F	GAGGCGTGCTCTACCTGTTTT
			R	CACAGACGGGTCGTAAAACTC
OCLN	OCLN	NM_002538	F	GAGGCTATGGAACTTCCCTTTT
			R	TAGCTACCAAAGCCACTTCCTC
RPLP0	RPLP0	NM_053275	F	CAGCTGATCAAGACTGGAGACA
			R	CACTTCAGGGTTGTAGATGCTG

* AN (Accession number).

**Table 2:** Descriptive and comparative summaries of the human BBB in vitro model development and processing: original 12-well TW model and the miniaturized and automated 96 TW system replicates.

BBB In Vitro System	Original 12 TW Model	96 TW Systems	
Brand	Corning	Falcon	Corning
Cell filter growth area	1.13 cm^2^	0.0804 cm^2^	0.143 cm^2^
Manual cell seeding ratio number (PCs well//ECs filter)	ECs 80,000//PCs 50,000	ECs 18,000//PCs 15,000	ECs > 15,000//PCs 15,000
Automated cell seeding ratio (PCs well//ECs filter)	-	ECs 18,000//PCs 15,000	ECs 22,000//PCs 15,000
Filters adaptation for cell visualization by Confocal Microscopy	Cut and placed in cover slip	96 TW ready for automation	96 TW ready for automation
Bottom plate adapted for cell visualization	Yes	No	Yes
Permeability/staining assays	Manually	Automated	Automated

## Data Availability

The datasets used and/or analyzed during the current study are available from the corresponding author upon request.
